# The regulation of artificial intelligence for health in Brazil begins with the General Personal Data Protection Law

**DOI:** 10.11606/s1518-8787.2022056004461

**Published:** 2022-08-22

**Authors:** Daniel de Araujo Dourado, Fernando Mussa Abujamra Aith

**Affiliations:** I Universidade de São Paulo Centro de Pesquisa em Direito Sanitário São Paulo SP Brasil Universidade de São Paulo. Centro de Pesquisa em Direito Sanitário. São Paulo, SP, Brasil; II Universidade de São Paulo Faculdade de Medicina Programa de Pós-Graduação em Saúde Coletiva São Paulo SP Brasil Universidade de São Paulo. Faculdade de Medicina. Programa de Pós-Graduação em Saúde Coletiva. São Paulo, SP, Brasil; III Universidade de São Paulo Faculdade de Saúde Pública Departamento de Política São Paulo SP Brasil Universidade de São Paulo. Faculdade de Saúde Pública. Departamento de Política, Gestão e Saúde. São Paulo, SP, Brasil

**Keywords:** Health Services Research, Artificial Intelligence, legislation & jurisprudence, Machine Learning, Health Law

## Abstract

Artificial intelligence develops rapidly and health is one of the areas where new technologies in this field are most promising. The use of artificial intelligence can modify the way health care and self-care are provided, besides influencing the organization of health systems. Therefore, the regulation of artificial intelligence in healthcare is an emerging and essential topic. Specific laws and regulations are being developed around the world. In Brazil, the starting point of this regulation is the *Lei Geral de Proteção de Dados Pessoais* (LGPD – General Personal Data Protection Law), which recognizes the right to explanation and review of automated decisions. Discussing the scope of this right is needed, considering the necessary instrumentalization of transparency in the use of artificial intelligence for health and the currently existing limits, such as the black-box system inherent to algorithms and the trade-off between explainability and accuracy of automated systems.

## INTRODUCTION

Artificial intelligence (AI) is beginning to change the world as we know it and is one of the most promising technologies for the health area. In the coming years, the use of AI in healthcare, particularly the deep learning subtype^1^, may significantly affect clinical practice, the management of health systems, and the relationship between patients and the healthcare network, by allowing them to process their own data to promote health^2^. Digital health will transform the structure of health services and national health systems, with great potential to improve quality and reduce costs in care^3^.

Therefore, AI regulation is now an essential issue in the health area. As well as every intervention that affects health, the incorporation of these new technologies needs to be stimulated while a regulatory structure, capable of ensuring that their use is entirely to the benefit of humans, is organized. AI systems must have proven quality and safety. Actions and services that have always been provided primarily by people begin to be heavily influenced and even performed by automated systems and it is a scenario that challenges basic premises of health regulation^4^.

Until the beginning of 2022, no specific guidelines or laws existed yet to regulate the use of artificial intelligence in health care^5^. This discussion is in progress in many countries and international bodies, and in Brazil, it starts with the *Lei Geral de Proteção de Dados Pessoais* (LGPD – General Personal Data Protection Law) (Federal Law no. 13,709/2018), which establishes the right to explanation and review of automated decisions in the Brazilian legal system. This is the normative expression of the principle of algorithmic transparency, which is central to the regulation of AI systems.

This article aims to discuss the scope of the right to explanation and review of automated decisions in AI regulation in health in Brazil starting from the LGPD, considering the international discussion on the topic and the currently existing limits for explainable AI in the health area.

### Principles for the Regulation of Artificial Intelligence in Health

Traditional AI, used since the 1950s, is different from the latest machine learning and deep learning techniques, which represent the great regulatory challenge. Machine learning is a type of AI that allows computers to learn automatically, without an explicit programming. Deep learning is a subtype of machine learning that works with a class of algorithms that uses models inspired by the central nervous system of living organisms, which are called artificial neural networks.

Deep learning algorithms can learn extremely complex relations to recognize patterns, therefore, they can make clinically relevant predictions from complex and heterogeneous data from medical records, clinical imaging, sensor continuous monitoring, as well as genomic data^6^. Different from traditional regulatory objects, such as medicines and medical devices, these “self-taught” algorithms are constantly changing. The ability of those systems to learn from real-world experience (training) and continuously improve their performance (adaptation) makes these technologies unique. Regulating them is like hitting a continuously moving target.

Algorithmic regulation has become a relevant concern in legal systems worldwide and is currently under construction in different countries and international bodies. Binding regulatory instruments (laws) are few and have been focusing mainly on data privacy. In other respects, regulation begins to be structured by codes of conduct and non-binding guidelines (soft law) created by government bodies, expert councils that advice public entities, research institutes, and private companies^7^.

The World Health Organization aims convergence to guide governments and other international bodies on the use of AI in health. Thus, based on general ethical principles for the development of AI and the incorporation of elements of bioethics and the current health regulation, we point out six key principles for the regulation of AI systems in health: 1) autonomy; 2) non-maleficence/beneficence; 3) transparency; 4) responsibility; 5) equity; 6) responsiveness/sustainability^5^. These principles are interconnected, without hierarchy between them, and need to be instrumentalized together.

### Algorithmic Transparency and the Right to Explanation

Transparency is the ethical principle most frequently found in general guidelines for the use of AI^7^ and is a key principle for AI in health. Transparency means that enough information about AI technologies is documented prior to their implementation, in order to facilitate public consultation and the understanding of how they work in the real world. These systems are expected to be intelligible and explainable to developers, health professionals, patients, users, and regulators, according to the ability of each group, and even each individual, to understand.

Instrumentalizing algorithmic transparency is necessary for other key principles for the use of AI in healthcare to be effective^8^: the protection of human autonomy (in order to ensure that people remain in control of health systems and medical decisions), safety and efficacy regulatory requirements (ensuring that AI will not harm people but promote well-being), accountability in the use of AI technologies^9^, and the search for equity (promoting social inclusion and ensuring that algorithms will not reproduce any kind of prejudice and discrimination). The expression of all these principles requires transparency of AI systems.

Nowadays, the main mechanism to express algorithmic transparency is the right to explanation of automated decisions, which is considered a fundamental element in algorithmic regulation. This concept has been consolidating since the drafting of the European General Data Protection Regulation (GDPR), in force since May 2018. The data subject shall have “the right to obtain human intervention, to express his or her point of view, to obtain an explanation of the decision reached after such assessment and to challenge the decision.” That is, besides receiving an intelligible explanation, the data subject also has the right to be heard, to question, and to request review of automated decisions. This is called “algorithmic due process”^10^.

Thus, the right to explanation regards the recognition that everyone shall be guaranteed the right to know how AI decisions that affect their lives are made. Since the publication of the GDPR—even before it came into force—the existence and scope of the right to explanation of automated decisions has been intensely discussed. As an AI algorithm can use numerous variables to reach a certain result, the complex mathematical representation is, in most cases, unintelligible to humans; thus, algorithms are commonly called black-box systems^11^.

The discussion is currently divided into two interpretations: on the one hand, those who advocate the feasibility and scope of the right to explanation only with regard to the overall system functionality, rather than specific decisions and individual circumstances^12^, and on the other hand, those who understand that the explanation shall also include specific decisions, with transparency limited only by the intrinsic black-box algorithmic system^13^.

The right to explanation is important as it gives patients the possibility to understand the logic of automated decisions that affect their health care. Such concern shall be increasingly present in several clinical situations. Nowadays, for example, deep learning algorithms that are able to define criteria for organ transplants, such as allocation, correspondence between donor and recipient, and chances of survival of transplant patients, already exist^14^. Soon these algorithms can be used for this purpose and cause differences in the order of transplant waiting lists in comparison with those based on clinical criteria made only by humans. The right to explanation is related to human dignity and this type of decision can not be made based only on black-box systems.

### The Right to Explanation from the General Personal Data Protection Law

Brazil enacted the LGPD in August 2018 and it entered into force in September 2020 (provisions on administrative sanctions only entered into force in August 2021). The LGPD is essentially devoted to personal data privacy and does not specifically address AI regulation,the terms “artificial intelligence” and “algorithm” do not even appear in the text. However, as it was openly inspired by the European GDPR and incorporated much of its rationality, this law introduces in Brazil the right to explanation and review of automated decisions.

The right to review of automated decisions is described in Article 20 of the LGPD, which grants data subjects the right to request review of decisions that affect their interests made based only on the automated processing of personal data, such as the GDPR. However, different from the European law, this Brazilian law does not provide the right not to be subject to exclusively automated decisions or to obtain human intervention in the case of a review. The original draft approved by the National Congress of Brazil provided the right of data subjects to request review of automated decisions “by a natural person”, but the provision was amended by a provisional measure that later became a law. Although Brazil excluded the requirement of human supervision from the LGPD, no fence prevents it to be required in non-statutory regulations.

The right to explanation does not appear in the text of the law (such as in the GDPR), but stems from the systematic interpretation of the LGPD along with constitutional provisions and consumer protection legislation^15^. Brazilian law guarantees to all those affected by automated decisions the right to obtain clear and adequate information on the criteria and procedures used. This is the expression of the principle of transparency, which can only be guaranteed by explanation.

The LGPD protects trade and industrial secrets in this and several other provisions, so that this consideration shall be made in non-statutory regulations and even in the analysis of specific cases. This trade secret protection may seem a way to promote the algorithm-based business model, but it must necessarily be weighed with the right to explanation of automated decisions in order to observe the ethical principles of using AI in line with human rights. The law itself provides an audit in case of suspected discrimination.

The rights to explanation and review of decisions of AI systems are necessarily linked and need to be understood together. Based on the European model, the configuration of these rights in Brazil still needs regulation and future doctrinal and jurisprudential elaboration, as it may occur in other countries.

### The Challenge of Explainable Artificial Intelligence in Health

The right to explanation is linked to the limits of algorithmic transparency. AI system transparency focuses mainly on the process, that is, it allows people to understand how algorithms are developed and implemented in general terms. It may eventually include factors of a specific prediction or decision, but it does not usually share codes or datasets.

Therefore, the existence of some opacity is inevitable. This opacity is related to the black-box system, due to the complexity of the systems, but an opacity (intentionally) imposed by corporate or state secrecy also exists, as sharing specific codes or datasets may expose trade secrets or disclose sensitive user data. The opacity may also be due to the users’ “technological illiteracy”^16^.

In this sense, an explanation providing the entire decision-making process of a system is neither feasible nor necessary. This explanation is essential in situations where some failure needs to be detected in a specific part of the system, especially when algorithms are increasingly being used to make recommendations or decisions currently subject to human discretion. However, an explanation shall respond to one of the following points^17^: 1) main decision factors: showing important factors for an AI prediction, preferably ordered by significance; 2) determining decision factors: clarifying factors that decisively affect results; 3) divergent results: explaining why two similar cases may give different results^18^.

The field of explainable AI is rapidly expanding. Nowadays, companies, standards bodies, non-profit organizations, and public institutions are developing much technical research to create AI systems that can explain their predictions. Designing systems to provide explanation is complex and expensive if they are developed with the possibility of providing a certain type of explanation (“inherent explainability”) and especially if the explanation comes after the algorithmic decision (“ *post hoc* explainability”)^19^. Therefore, the search for explainable models for high-risk areas, such as health care, has been driving research^20^.

However, the limits for explainable AI in health are quite relevant.

First, the existence of a trade-off between explainability and accuracy must be considered^21^. An explainable AI system, most of the time, needs to reduce solution variables to a set small enough to become accessible to human understanding. It may hinder the use of some systems in complex problems. Some deep learning models can accurately predict probabilities of clinical diagnoses but they are humanly incomprehensible. In this sense, a broader right to explanation, based on maximum transparency, may conflicts with the use of automated systems with high predictive accuracy.

Moreover, the techniques currently available for explainability are able to broadly describe how AI systems work in general, but they are very superficial or unreliable for individual decisions^22^. In practice, explanations can be very useful in global AI processes, such as model development and auditing, but are rarely informative about specific results given by algorithms.

Therefore, this current lack of transparency may persist, at least for some time. To some extent, opacity is a usual feature in clinical activity. Medicine traditionally adopts practices that involve mechanisms that are not fully understood but that continue to be widely used due to their proven effects, such as many medications. We must recognized the obstacles to develop an explainable AI in health and carefully consider them when elaborating regulatory mechanisms that consider the limits of explainability and consequently the scope of the right to explanation and review of automated decisions in health.

## CONCLUSION

The application of the right to explanation in health shall include specific complexities of AI regulation for clinical use. As this right is now present in Brazilian legislation, regulatory bodies are responsible to limit its scope and mechanisms so that it can be instrumentalized. Besides the actions of the *Autoridade Nacional de Proteção de Dados* (ANPD – National Data Protection Authority), other regulatory bodies, such as the *Agência Nacional de Vigilância Sanitária* (ANVISA – Brazilian Health Regulatory Agency), and regulatory authorities of regulated professions, such as medical councils, shall intervene.

The exercise of the right to explanation in health depends on the creation of mechanisms for the development of explainable AI systems and on the recognition of the limits of algorithm explainability. The scope of this right must be defined based on criteria to be elaborated by regulatory authorities and need to be widely discussed with society. This discussion is just beginning.

## References

[1] Obermeyer Z, Emanuel EJ. Predicting the future: big data, machine learning, and clinical medicine. N Engl J Med. 2016;375(13):1216-9. https://doi.org/10.1056/NEJMp1606181 10.1056/NEJMp1606181 PMC5070532 27682033

[2] Topol EJ. High-performance medicine: the convergence of human and artificial intelligence. Nat Med. 2019;25:44-56. https://doi.org/10.1038/s41591-018-0300-7 10.1038/s41591-018-0300-7 30617339

[3] World Health Organization. mHealth: use of appropriate digital technologies for public health: report by the director-general. In: 71. WHO Assembly; 2018 Mar 26; Geneva, Switzerland. Geneva (CH): WHO; 2018 [cited 2022 Feb 1]. Available from: https://apps.who.int/gb/ebwha/pdf_files/WHA71/A71_20-en.pdf

[4] Richman B. Health regulation for the digital age: correcting the mismatch. N Engl J Med. 2018;379(18):1694-5. https://doi.org/10.1056/NEJMp1806848 10.1056/NEJMp1806848 30380380

[5] World Health Organization. Ethics and governance of artificial intelligence for health: WHO guidance. Geneva (CH): WHO; 2021 [cited 2022 Feb 1]. Available from: https://www.who.int/publications/i/item/9789240029200

[6] Rajkomar A, Dean J, Kohane I. Machine learning in medicine. N Engl J Med. 2019;380(14):1347-58. https://doi.org/10.1056/NEJMra1814259 10.1056/NEJMra1814259 30943338

[7] Jobin A, Ienca M, Vayena E. The global landscape of AI ethics guidelines. Nat Mach Intell. 2019;1:389-99. https://doi.org/10.1038/s42256-019-0088-2

[8] Watson DS, Krutzina J, Bruce IN, Griffiths CE, McInnes IB, Barnes MR et al. Clinical applications of machine learning algorithms: beyond the black box. BMJ. 2019;364:l886. https://doi.org/10.1136/bmj.l886 10.1136/bmj.l886 30862612

[9] Vayena E, Blasimme A, Cohen IG. Machine learning in medicine: addressing ethical challenges. PLoS Med. 2018;15(11):e1002689. https://doi.org/10.1371/journal.pmed.1002689 10.1371/journal.pmed.1002689 PMC6219763 30399149

[10] Kaminski ME. The right to explanation, explained. Berkeley Technol Law J. 2019;34(1):189-218. https://doi.org/10.15779/Z38TD9N83H

[11] Price WN II. Medical malpractice and black-box medicine. In: Cohen IG, Lynch HF, Vayena E, Gasser U, editors. Big data, health law, and Bioethics. Cambridge (UK): Cambridge University Press; 2018. Chapter 20; p. 295-306. https://doi.org/10.1017/9781108147972.027

[12] Wachter S, Mittelstadt B, Floridi L. Why a right to explanation of automated decision-making does not exist in the General Data Protection Regulation. Int Data Priv Law. 2017;7(2):76-99. https://doi.org/10.1093/idpl/ipx005

[13] Selbst AD, Powles J. Meaningful information and the right to explanation. Int Data Priv Law. 2017;7(4):233-42. https://doi.org/10.1093/idpl/ipx022

[14] Khorsandi SE, Hardgrave HJ, Osborn T, Klutts G, Nigh J, Spencer-Cole RT, et al. Artificial intelligence in liver transplantation. Transplant Proc. 2021;53(10):2939-44. https://doi.org/10.1016/j.transproceed.2021.09.045 10.1016/j.transproceed.2021.09.045 34740449

[15] Monteiro R, Machado C, Silva L. The right to explanation in Brazilian data protection law. RIDDN [Internet]. 2021 [cited 2022 Feb 1];7(1):119-136. Available from: https://ojs.imodev.org/?journal=RIDDN&page=article&op=view&path%5B%5D=406

[16] Burrell J. How the machine ‘thinks’: understanding opacity in machine learning algorithms. Big Data Soc. 2016;3(1):1-12. https://doi.org/10.1177/2053951715622512

[17] Organisation for Economic Co-Operation and Development. Artificial Intelligence in society. Paris (FR): Éditions OCDE; 2019.

[18] Doshi-Velez F, Kortz M, Budish R, Bavitz C, Gershman S, O’Brien D, et al. Accountability of AI under the law: the role of explanation. arXiv:1711.01134v3 [Preprint]. 2017 [cited 2017 Nov 3; revised 2019 Dec 20]. Available from: https://arxiv.org/abs/1711.01134v3

[19] Lipton ZC. The mythos of model interpretability: in machine learning, the concept of interpretability is both important and slippery. Queue. 2018;16(3):31-57. https://doi.org/10.1145/3236386.3241340

[20] Holzinger A, Biemann C, Pattichis CS, Kell DB. What do we need to build explainable AI systems for the medical domain? arXiv:1712.09923v1 [Preprint]. 2017 [cited 2017 Dec 28]. Available from: https://arxiv.org/abs/1712.09923v1

[21] London AJ. Artificial Intelligence and black-box medical decisions: accuracy versus explainability. Hastings Cent Rep. 2019;49(1):15-21. https://doi.org/10.1002/hast.973 10.1002/hast.973 30790315

[22] Ghassemi M, Oakden-Rayner Luke, Beam AL. The false hope of current approaches to explainable artificial intelligence in health care. Lancet Digit Health. 2021;3(11):e745-50. https://doi.org/10.1016/S2589-7500(21)00208-9 10.1016/S2589-7500(21)00208-9 34711379

