# Supplementary material for: The regulation of artificial intelligence for health in Brazil begins with the General Personal Data Protection Law
Source: Rev Saude Publica. 2022 Aug 22;56:80. doi: 10.11606/s1518-8787.2022056004461 (PMC9423092; doi:10.11606/s1518-8787.2022056004461)
Supplement: Translation

# Translation: A regulação da inteligência artificial na saúde no Brasil começa com a Lei Geral de Proteção de Dados Pessoais

**Authors:** Daniel de Araujo Dourado, Fernando Mussa Abujamra Aith

**Affiliations:** I Brasil Universidade de São Paulo. Centro de Pesquisa em Direito Sanitário. São Paulo, SP, Brasil; II Brasil Universidade de São Paulo. Faculdade de Medicina. Programa de Pós-Graduação em Saúde Coletiva. São Paulo, SP, Brasil; III Brasil Universidade de São Paulo. Faculdade de Saúde Pública. Departamento de Política, Gestão e Saúde. São Paulo, SP, Brasil

**Keywords:** Pesquisa sobre Serviços de Saúde, Inteligência Artificial, legislação & jurisprudência, Aprendizado de Máquina, Direito Sanitário

## Abstract

A inteligência artificial se desenvolve rapidamente e a saúde é uma das áreas em que as novas tecnologias desse campo são mais promissoras. O uso de inteligência artificial tem potencial para modificar a forma de prestação da assistência à saúde e do autocuidado, além de influenciar a organização dos sistemas de saúde. Por isso, a regulação da inteligência artificial na saúde é um tema emergente e essencial. Leis e normas específicas são elaboradas em todo o mundo. No Brasil, o marco inicial dessa regulação é a Lei Geral de Proteção de Dados Pessoais, a partir do reconhecimento do direito à explicação e à revisão de decisões automatizadas. É preciso debater a abrangência desse direito, considerando a necessária instrumentalização da transparência no uso da inteligência artificial na saúde e os limites atualmente existentes, como a dimensão caixa-preta inerente aos algoritmos e o *trade-off* existente entre explicabilidade e precisão dos sistemas automatizados.

## INTRODUÇÃO

A inteligência artificial (IA) começa a mudar o mundo como conhecemos e é uma das tecnologias mais promissoras para a área da saúde. O uso da IA na saúde, particularmente o subtipo *deep learning* ^1^, deve ter impacto significativo nos próximos anos na prática clínica, na gestão de sistemas de saúde e na relação entre os pacientes e a rede assistencial – ao permitir que eles processem seus próprios dados para promover a saúde^2^. A Saúde Digital vai transformar a estrutura dos serviços de saúde e dos sistemas nacionais de saúde, com grande potencial para melhoria de qualidade e redução de custos na assistência^3^.

Por isso, a regulação da IA é hoje tema essencial no campo da saúde. Como toda intervenção que afeta a saúde, a incorporação dessas novas tecnologias precisa ser estimulada ao mesmo tempo em que se organiza uma estrutura regulatória capaz de assegurar que seu uso seja impreterivelmente em benefício dos seres humanos. Os sistemas de IA devem ter qualidade e segurança comprovadas e deve-se reconhecer que as ações e serviços, que sempre foram prestados principalmente por pessoas, começam a ser fortemente influenciados e até mesmo executados por sistemas automatizados. É um cenário que desafia pressupostos básicos da regulação em saúde^4^.

Até o início do ano de 2022, ainda não havia diretrizes ou leis específicas para regular o uso da inteligência artificial na assistência à saúde^5^. Esse debate está em andamento em muitos países e entidades internacionais e chega ao Brasil com a Lei Geral de Proteção de Dados Pessoais (LGPD – Lei Federal n^o^13.709/2018), passando a vigorar e instituindo o direito à explicação e à revisão de decisões automatizadas no ordenamento jurídico brasileiro. Trata-se da expressão normativa do princípio da transparência de algoritmos, que é ponto central na regulação dos sistemas de IA.

Este artigo tem por objetivo discutir a abrangência do direito à explicação e à revisão de decisões automatizadas na regulação da IA na saúde no Brasil a partir do marco da LGPD, considerando o debate internacional sobre o tema os limites atualmente existentes para IA explicável na área da saúde.

### Princípios para Regulação da Inteligência Artificial na Saúde

É importante diferenciar a IA tradicional – usada desde a década de 1950 – das técnicas mais recentes de *machine learning* e *deep learning* , porque são elas que representam o grande desafio regulatório. *Machine learning* é um tipo de IA que permite aos computadores aprender automaticamente, sem programação explicíta. *Deep learning* é um subtipo de *machine learning* que consiste em uma classe de algoritmos que usam modelos inspirados no sistema nervoso central de organismos vivos, denominados redes neurais artificiais.

Os algoritmos de *deep learning* podem aprender relações extremamente complexas para reconhecimento de padrões e, por isso, podem ajudar a fazer previsões clinicamente relevantes a partir de dados complexos e heterogêneos, gerados em atendimento clínico, como registros médicos, imagens clínicas, dados de monitoramento contínuo de sensores e dados genômicos^6^. Diferentemente dos objetos da regulação tradicional, como medicamentos e dispositivos médicos, esses algoritmos “autodidatas” estão em constante mudança. A capacidade desses sistemas de aprender com a experiência do mundo real (treinamento) e melhorar continuamente o desempenho (adaptação) torna essas tecnologias únicas. Regulá-los é como atingir um alvo em contínuo movimento.

A regulação de algoritmos tem se tornado uma preocupação relevante nos sistemas jurídicos em todo o mundo e, atualmente, está em construção em diferentes países e órgãos internacionais. Os instrumentos normativos vinculantes (leis) são escassos e têm se concentrado principalmente no campo da privacidade dos dados. Nos demais aspectos, a abordagem regulatória começa a se estruturar por meio de códigos de conduta e diretrizes não vinculantes ( *soft law* ), produzidos por entidades governamentais, conselhos de especialistas para assessoramento de entes públicos, institutos de pesquisa e empresas privadas^7^.

A Organização Mundial da Saúde tem buscado convergência para orientar os governos e as demais entidades internacionais no uso da IA na saúde. Nesse sentido, a partir de princípios éticos gerais para desenvolvimento de IA e da incorporação de elementos da bioética e da atual regulação em saúde, identificam-se seis princípios-chave para a regulação dos sistemas de IA na saúde: 1) autonomia; 2) não-maleficência/beneficência; 3) transparência; 4) responsabilidade; 5) equidade; 6) responsividade/sustentabilidade^5^. Esses princípios estão interligados, sem hierarquia entre eles, e precisam ser instrumentalizados conjuntamente.

### Transparência Algorítmica e Direito à Explicação

A transparência é o princípio ético mais frequentemente encontrado nos códigos de diretrizes gerais para o uso da IA^7^e é um princípio-chave para a IA na saúde. Transparência significa que informações suficientes sobre as tecnologias de IA sejam documentadas antes da implantação de modo a facilitar consulta pública e entendimento sobre seu funcionamento no mundo real. Espera-se que os sistemas sejam inteligíveis e explicáveis para desenvolvedores, profissionais de saúde, pacientes, usuários e reguladores, de acordo com a capacidade de compreensão de cada grupo e mesmo de cada indivíduo.

Instrumentalizar a transparência dos algoritmos é necessário para que outros princípios-chave do uso da IA na saúde tenham eficácia^8^: a proteção da autonomia humana, para assegurar que as pessoas permaneçam no controle dos sistemas de saúde e das decisões médicas; os requisitos regulatórios de segurança e eficácia, que garantam que a IA não prejudique as pessoas e promova o bem-estar; a prestação de contas ( *accountability* ) no uso de tecnologias de IA^9^; a busca de equidade, que promova inclusão social e assegure que algoritmos não reproduzam qualquer tipo de preconceito e discriminação. A expressão de todos esses princípios pressupõe transparência dos sistemas de IA.

Atualmente, o principal mecanismo para expressão da transparência algorítmica tem sido o direito à explicação ( *right to explanation* ) sobre decisões automatizadas, considerado elemento fundamental na regulação de algoritmos. Esse conceito vem se consolidando desde a elaboração do Regulamento Geral sobre a Proteção de Dados da União Europeia (mais comumente identificado pela sigla GDPR, do inglês *General Data Protection Regulation* ), em vigor desde maio de 2018. O titular dos dados deve ter “o direito de obter a intervenção humana, de manifestar o seu ponto de vista, de obter uma explicação sobre a decisão tomada na sequência dessa avaliação e de contestar a decisão”. Ou seja, além de receber uma explicação inteligível, cria-se o direito à oportunidade de ser ouvido, de questionar e pedir revisão da decisão automatizada. É o que vem sendo chamado de “devido processo algorítmico”^10^.

Em suma, o direito à explicação diz respeito ao reconhecimento de que deve ser assegurado a todas as pessoas o direito de saber como são tomadas as decisões baseadas em IA que afetam suas vidas. Desde a publicação do GDPR, mesmo antes de sua entrada em vigor, há intensas discussões sobre a existência e o alcance do direito à explicação nas decisões automatizadas. Como um algoritmo de IA pode usar inúmeras variáveis para chegar a um determinado resultado, a forma complexa de representação matemática é, na maior parte das vezes, ininteligível para os humanos – por isso os algoritmos são comumente chamados de sistemas de “caixa-preta”^11^.

Em linhas gerais, o debate atualmente se divide em duas interpretações. Por um lado, os que defendem a viabilidade e o escopo do direito à explicação apenas no que diz respeito à funcionalidade geral do sistema, ao invés de sobre decisões específicas e circunstâncias individuais^12^. Por outro lado, há o entendimento de que a explicação também deve incluir decisões específicas, com transparência limitada apenas pela dimensão intrinsecamente de caixa-preta dos algoritmos^13^.

A importância do direito à explicação é dar aos pacientes a possibilidade de compreender a lógica de decisões automatizadas que impactem condutas tomadas nos seus cuidados de saúde. Tal preocupação deve estar cada vez mais presente em diversas situações clínicas. Por exemplo, atualmente já existem algoritmos de *deep learning* capazes de definir critérios para transplantes de órgãos, como alocação, correspondência entre doador e receptor e de previsão de sobrevida dos pacientes transplantados^14^. É bem possível que em breve esses algoritmos possam ser usados para essa finalidade e que haja diferenças de ordem nas filas de transplantes em comparação com as definidas por critérios clínicos feitas apenas por humanos. O direito à explicação relaciona-se à dignidade humana e não será aceitável que decisões dessa natureza sejam tomadas apenas com base em sistemas do tipo caixa-preta.

### Direito à Explicação a Partir da Lei Geral de Proteção de Dados Pessoais

No Brasil, a LGPD foi promulgada em agosto de 2018 e entrou em vigor em setembro de 2020 (os dispositivos referentes às sanções administrativas só entraram em vigor em agosto de 2021). Fazendo jus a seu nome, a LGPD é essencialmente dedicada à privacidade de dados pessoais e não trata especificamente da regulação de IA – os termos “inteligência artificial” e “algoritmo” sequer aparecem no texto. Mas como foi abertamente inspirada no GDPR europeu e incorporou muito da sua racionalidade, a lei introduz no direito brasileiro o direito à explicação e à revisão de decisões automatizadas.

O direito à revisão de decisões automatizadas está definido explicitamente no texto do art. 20 da LGPD, que concede ao titular o direito a solicitar a revisão de decisões tomadas unicamente com base em tratamento automatizado de dados pessoais que afetem seus interesses, a exemplo do GDPR. Diferentemente da legislação europeia, a lei brasileira não prevê o direito de não se sujeitar a decisão exclusivamente automatizada e nem de obter intervenção humana em caso de revisão. A redação original aprovada pelo Congresso Nacional previa o direito do titular de solicitar revisão de decisões automatizadas “por pessoa natural”, mas o dispositivo foi alterado por medida provisória, posteriormente convertida em lei. Vale ressaltar que, embora a obrigação de supervisão humana tenha sido excluída da LGPD, não há vedação para que essa exigência seja feita na regulamentação infralegal.

O direito à explicação não aparece textualmente (assim como no GDPR), mas decorre da interpretação sistemática da própria LGPD em conjunto com dispositivos constitucionais e da legislação de proteção ao consumidor^15^. A lei brasileira garante a todo aquele afetado por decisões automatizadas o direito a obter informações claras e adequadas a respeito dos critérios e dos procedimentos utilizados. Isso é a expressão do princípio da transparência, que só pode ser garantido por meio da explicação.

A LGPD ressalva os segredos comercial e industrial nesse e em diversos outros dispositivos, de modo que essa ponderação deverá ser feita na regulamentação infralegal e mesmo na análise de casos concretos. A proteção dos segredos de negócio na LGPD pode ser vista como uma forma de fomentar o modelo empresarial baseado em algoritmos, mas deve necessariamente ser sopesada com o direito à explicação sobre decisões automatizadas a fim de observar os princípios éticos de uso da IA em harmonia com os direitos humanos. A própria lei sinaliza nesse sentido ao prever a realização de auditoria em caso de suspeita de discriminação.

Os direitos à explicação e à revisão de decisões de sistemas de IA estão necessariamente vinculados e precisam ser compreendidos conjuntamente. Assim como deve acontecer em outros países, com base no modelo europeu, a configuração desses direitos no Brasil ainda necessita de regulamentação e de futura elaboração doutrinária e jurisprudencial.

### O Desafio da Inteligência Artificial Explicável na Saúde

O direito à explicação está vinculado aos limites da transparência algorítmica. A transparência de um sistema de IA se concentra principalmente no processo, ou seja, trata-se de permitir que as pessoas entendam como os algoritmos são desenvolvidos e implantados em termos gerais. Eventualmente pode incluir elementos sobre fatores de uma previsão ou decisão específica, mas em regra não inclui o compartilhamento de códigos ou conjuntos de dados.

Logo, a existência de alguma opacidade é inevitável. Essa opacidade tem relação com a dimensão de caixa-preta, pela complexidade dos sistemas, mas não só. Há também opacidade imposta por sigilo corporativo ou de Estado (intencional), pois o compartilhamento de códigos ou conjuntos de dados específicos pode revelar segredos comerciais ou divulgar dados confidenciais do usuário. E opacidade decorrente do próprio “analfabetismo técnico” dos usuários^16^.

Nesse sentido, não é viável nem necessário que uma explicação forneça todo o processo de tomada de decisão do sistema. A explicação é essencial para situações em que alguma falha precisa ser determinada em uma instância específica do sistema, sobretudo quando algoritmos começam a ser cada vez mais usados para fazer recomendações ou decisões atualmente sujeitas à discrição humana. Mas é suficiente que a explicação consiga responder a um dos seguintes pontos^17^: 1) principais fatores de decisão: indicar os fatores importantes para uma previsão de IA, preferencialmente ordenados por significância; 2) fatores determinantes de decisão: esclarecer fatores que afetam decisivamente o resultado; 3) resultados divergentes: explicar por que dois casos de aparência semelhante podem resultar em resultados diferentes^18^.

O campo de IA explicável está em acelerada expansão. Atualmente há muitas pesquisas técnicas em desenvolvimento por empresas, órgãos de normalização, organizações sem fins lucrativos e instituições públicas com intuito de criar sistemas de IA que possam explicar suas previsões. Projetar um sistema para fornecer explicações é complexo e caro, tanto se forem desenvolvidos prevendo a possibilidade de fornecer um certo tipo de explicação (“explicabilidade inerente”) quanto principalmente se explicações forem buscadas após a decisão algorítmica (“explicabilidade *post hoc* ”)^19^. Por isso, as pesquisas têm sido impulsionadas sobretudo na busca de modelos explicáveis para áreas de alto risco, como é o caso do uso na assistência à saúde^20^.

No entanto, os limites para a IA explicável na saúde são bastante relevantes.

Primeiramente, é preciso considerar que existe um *trade-off* entre explicabilidade e precisão^21^. Para que um sistema de IA seja explicável, na maioria das vezes é necessária uma redução das variáveis da solução a um conjunto pequeno o suficiente para que fique acessível ao entendimento humano. Isso pode inviabilizar o uso de alguns sistemas em problemas complexos. Alguns modelos de *deep learning* podem prever probabilidades de diagnósticos clínicos com precisão, mas serem humanamente incompreensíveis. Nesse sentido, um direito à explicação mais amplo, baseado na máxima transparência, pode ser incompatível com o uso de sistemas automatizados que busquem alta acurácia preditiva.

Além disso, as técnicas atualmente disponíveis para explicabilidade são capazes de oferecer descrições amplas de como o sistema de IA funciona em sentido geral, mas são muito superficiais ou não confiáveis para decisões individuais^22^. Na prática, as explicações podem ser muito úteis em processos globais de IA, como desenvolvimento de modelo e auditoria, mas raramente são informativas com relação a resultados específicos dados pelos algoritmos.

Portanto, a atual falta de transparência deve persistir, ao menos por algum tempo. Em certa medida, opacidade é uma característica usual na atividade clínica. A medicina tradicionalmente adota práticas que envolvem mecanismos não totalmente compreendidos, mas que continuam sendo amplamente usados, devido aos efeitos comprovados, como é o caso de muitos medicamentos. Os obstáculos para desenvolver uma IA explicável na saúde devem ser reconhecidos e bem ponderados na construção de mecanismos regulatórios que considerem os limites da explicabilidade e consequentemente a abrangência do direito à explicação e à revisão de decisões automatizadas na assistência à saúde.

## CONCLUSÃO

A aplicação do direito à explicação na saúde deverá contemplar complexidades específicas da regulação da IA para uso clínico. Partindo do entendimento de que esse direito está agora presente na legislação brasileira, caberá aos órgãos reguladores delimitar a sua amplitude e mecanismos para que possa ser instrumentalizado. Além da atuação da Autoridade Nacional de Proteção de Dados, deve haver intervenção de outros órgãos reguladores, como a Agência Nacional de Vigilância Sanitária e das autoridades reguladoras de profissões regulamentadas, como os conselhos de medicina.

O exercício do direito à explicação na saúde depende da criação de mecanismos para criação de sistemas de inteligência artificial explicáveis e do reconhecimento dos limites da explicabilidade de algoritmos. A abrangência desse direito deve ser definida a partir de critérios a serem elaborados por autoridades reguladoras e precisam ser amplamente discutidos com a sociedade. Esse debate está apenas começando.
